# Regional retrospective observational analysis of the impact of enhanced care teams on trauma morbidity and mortality outcomes

**DOI:** 10.1186/s13049-025-01481-5

**Published:** 2025-10-24

**Authors:** Christopher Smith, Ryan McHenry, Sarah Norman, Richard Hardern

**Affiliations:** 1 Great North Air Ambulance Service, Stockton-on-Tees, United Kingdom; 2 Northern Trauma Network, Sunderland, United Kingdom; 3 ScotSTAR, Scottish Ambulance Service, Paisley, United Kingdom; 4 Northern Deanery, Newcastle, United Kingdom

## Abstract

**Background:**

In the UK, prehospital enhanced care teams (ECT) including Ground Emergency Medical Services or Helicopter Emergency Medical Services are staffed by doctors and critical care paramedics. To date, it has remained unclear whether the advanced interventions that can be delivered by an ECT generate demonstrable benefit in patient outcome. This study compares the morbidity and mortality of injured patients who received ‘standard’ paramedic-only care with those who were attended to additionally, or exclusively, by an ECT, comprising Pre-Hospital Emergency Medicine consultant and critical care paramedic.

**Methods:**

In collaboration with the Northern Trauma Network, a retrospective analysis of Trauma Audit and Research Network (TARN) data and case note review of all severe trauma cases (Injury Severity Score ≥ 9) in Cumbria and the North East of England, between 1 January 2010 to 31 December 2022 was completed. Patients treated by the North East, North West ambulance service and Great North Air Ambulance Services were included. TARN records were used to calculate Ws statistics in ECT and non-ECT groups to provide a measure of mortality adjusted for case mix. Glasgow Outcome Scales were contrasted to evaluate morbidity.

**Results:**

1724 patients in the ECT group and 3327 in the non ECT group were studied. There was an association between ECT care and improved survival. The difference in observed and expected survival was + 69 in the ECT group and − 57 in the non-ECT group. The difference between the two groups’   Ws statistic was 5.33 (95% CI 3.63 to 7.03), equivalent to one extra survivor for every 19 patients treated by an ECT group. There was no significant difference in morbidity between the two groups.

**Conclusion:**

This study demonstrates a risk-adjusted significant mortality association in trauma patients, an additional 3.48 to 5.3 survivors per 100 severe (ISS ≥ 9) trauma casualties when treated by an ECT. This study details five key recommendations for future practice within HEMS. The authors encourage other ECT services to conduct further high-quality research.

**Clinical trial number:**

Not applicable.

## Introduction

The importance of administering critical care interventions promptly after injury has been discussed in the literature. Research has explored the impact of enhanced care teams (ECTs) on all trauma patients, noting differences in the baseline characteristics of patients treated by ECTs and those treated by non-ECTs [1]. Due to these differences and inadequate matching of the groups, several studies have failed to demonstrate a mortality benefit from ECT interventions [2–4].

This study represents a continuation of previous research, focusing on severely injured patients (Injury Severity Score [ISS] ≥ 9) predominantly (95%) from blunt mechanisms. It includes data from more patients, over a longer observation period (13 years), and from a broader geographical scope; increasing the precision of estimates derived from the data [5].

Our objective is to investigate differences in mortality and morbidity between patients who receive standard prehospital care and those who receive care exclusively from an ECT. More specifically, we aim to determine whether ECT care affects the likelihood of unexpected survival among patients with severe trauma.

## Methods

### Description of service

**Fig. 1:**
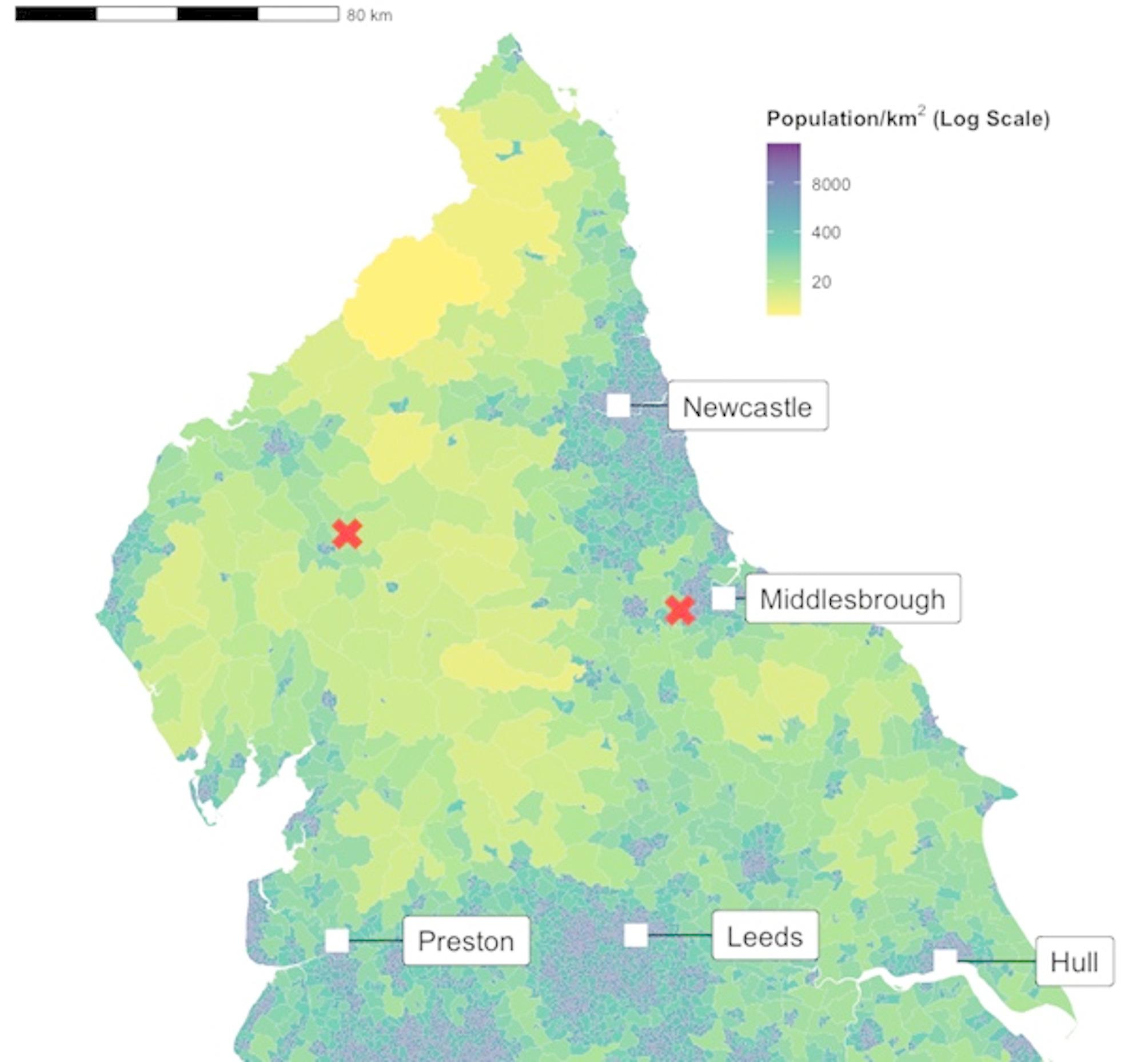
Map of the North of England demonstrating population density alongside Major Trauma Centre locations (boxes), and the location of Great North Air Ambulance Service bases (crosses)

The Great North Air Ambulance Service (GNAAS) operates two bases covering a population of four million individuals across 8,000 square miles in Northern England (Fig. 1). The service utilises two helicopters, which are replaced with rapid response vehicles (RRVs) during adverse weather conditions and low light, ensuring continuous coverage seven days a week from 08:00 to 20:00 hours. Over the past eight years, the RRV service has expanded to provide nighttime coverage every night between 20:00 and 08:00 hours in the North East and Thursday to Sunday nights in the Cumbria. GNAAS autonomously dispatches to incidents based on specific criteria with the objective of identifying patients who meet major trauma triage criteria from 08:00 to 20:00 hours. Outside of these hours, local ambulance control centres task the RRV. Trauma cases constitute 77% of GNAAS missions.

The Enhanced Care Team (ECT) consists of a critical care paramedic (CCP) and a Pre-Hospital Emergency Medicine (PHEM) consultant 90% of the time, and a CCP and a senior PHEM trainee in all other instances.

The authors chose mortality and morbidity as patient centred outcomes. The primary outcomes were defined as follows:

- Survival was assessed using the Ws statistic, that is comparison of observed survival with the expected survival based on TARN Probability of Survival (Ps17), after adjustment of case mix as is standard TARN methodology.
- Survival was recorded to hospital discharge.
- Injury related mortality was death within 30 days of admission. This was chosen as most trauma deaths occur in the initial weeks following trauma [6].
- Morbidity was classified using the Glasgow Outcome Scale.

Critical care procedures, anaesthesia and blood product transfusion, were considered to determine their influence of mortality and morbidity.

### Inclusion criteria and exclusion criteria

In collaboration with the Northern Trauma Network (NTN), the Trauma Audit Research Network (TARN) provided data for all patients (both adult and paediatric) who presented to hospitals in NTN during the operational hours of GNAAS, between 1st January 2010 and 31st December 2022.

The regional Major Trauma Centres (MTCs) are James Cook University Hospital, Middlesbrough, the Royal Victoria Infirmary, Newcastle, and the Royal Preston Hospital. All have similar survival rates according to TARN performance analysis and adhere to similar guidelines in the Emergency Department (ED).

To identify patients, most likely to benefit from ECT care, major trauma patients with Injury Severity Score (ISS) ≥ 9 were included in analysis who were included in the TARN database and met any of the following criteria:

► Hypotensive on ED arrival (systolic blood pressure (SBP) < 90mmHg).

► Transfused blood products prehospital or in the ED or both.

► Intubated prehospital or in the ED.

► Admitted directly to level 2 or 3 critical care from the ED.

► Transferred to the operating theatre within 4 h of ED arrival.

► Died in the first 24 h post-admission to hospital.

Patients were excluded from the study if they fulfilled any of the following criteria:

► Did not trigger the prehospital major trauma triage tool.

► Self-presented to hospital.

► Sustained an isolated neck of femur fracture or a single rib fracture.

► Were in traumatic cardiac arrest and were not conveyed to hospital i.e. died on scene (these patients are not included in the TARN database).

### Data sources

Age, gender, mechanism of injury, ISS, GCS, mortality and morbidity data were obtained from TARN. Records of interventions, blood products and death within the first 24 hours were obtained by a single reviewer from hospital and prehospital notes. ECT group patients were identified by cross-referencing TARN data with a separate prehospital database held by GNAAS. The study was registered with the receiving MTC base hospitals as a service evaluation; ethics approval was not required.

### Statistical analysis

The primary outcome was in-hospital mortality. The excess survivors per hundred patients (Ws) was calculated separately for the two groups.

TARN calculates the predicted outcome of each patient using the Ps17 (Probability of Survival) score. This is calculated using a logistic regression model including coefficients for patient characteristics such as age, sex, ISS, GCS and comorbidities. If, for example, a patient has a Ps17 of 0.7 then 70 out of every 100 patients with that profile would be expected to survive. The W statistic compares observed and predicted (from Ps17) mortality [7]:

$$\:W\:=\:\frac{actual\:number\:of\:survivors\:-\:predicted\:number\:of\:survivors}{number\:of\:patients/100}$$

This is then risk standardised (Ws) using the TARN standard (a predetermined weight is given to each of the Ps band) to allow direct comparison between hospitals by compensating for variation in case mix of injury severities. A positive Ws indicates more survivors than expected, and a negative score indicates fewer survivors than expected [8].

Baseline characteristics and critical care interventions were compared using the Bonett and Price test Table 1 for medians and the Wilson method for comparison of two proportions. The authors compared the outcome of the patients treated by ECTs and non-ECTs by comparing the Ws score of each.

Glasgow Outcome Scale (GOS) at three months were analysed for the cohort using similar methodology to previous literature [9] GOS was dichotomised to better outcomes (GOS 4–5) and worse outcomes (GOS 1–3), and the association between these outcomes, ECT presence and Ps17 score was assessed in univariate and multivariate logistic regression.

## Results

### Patients included in analyses

**Fig. 2:**
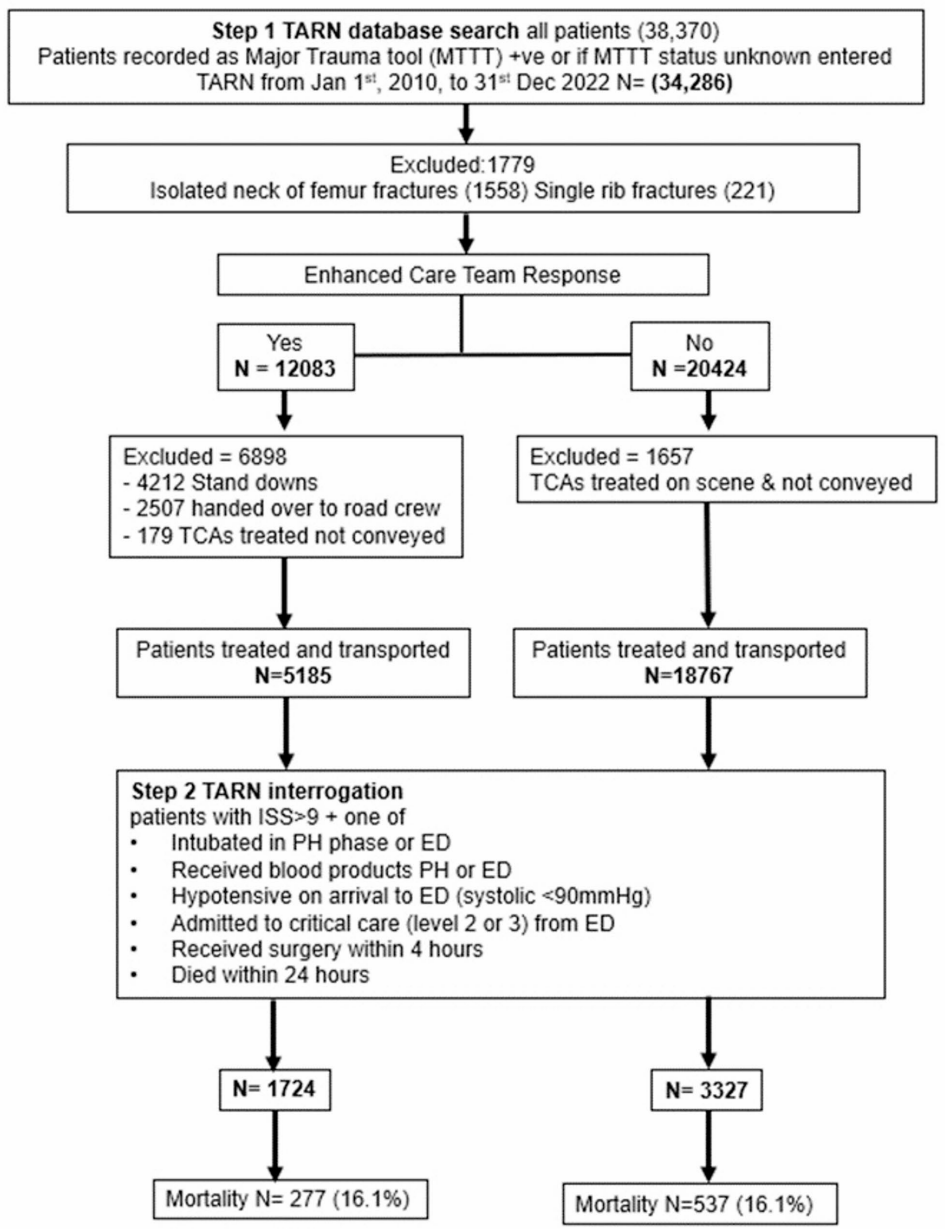
Study flow diagram

The TARN database included 38,370 potentially eligible patients from the 13-year study period (Fig. 2). Of these 1995 patients activated the Northern Trauma Network regional prehospital major trauma tool (MTT) to warrant Trauma Unit (TU) bypass or admission to an MTC. A further 32,291 did not have any MTT status recorded. 1779 were excluded because their injury was either an isolated fractured neck of femur or a single rib fracture.

12,083 of these patients triggered an ECT response and 20,424 did not. 6898 of the ECT responses were excluded from further analysis, leaving 5185 for further consideration. 1657 of the non-ECT group were excluded as they were treated on scene and not conveyed, leaving 18,767 for further consideration.

Of the initial 12,083 taskings in the ECT group, 1724 (4%) patients met the study inclusion criteria, 1571 (91%) of these received critical care interventions. The ambulance service treated and transported to hospital 18,767 trauma patients of whom; 3327 (17%) patients met the inclusion criteria. 1266 (38%) received critical care interventions on arrival at the Emergency Department.

### Baseline characteristics

**Table 1:** Baseline Characteristics

**Characteristic**	**Non-ECT**(***n*=3327)**	**ECT**(***n*=1724)**	**ECT – non ECT Difference of Median or Proportion (95% CI)**
**Demographics & injury severity**			
Male, count	2300 (69.1%)	1259 (73.0%)	0.04 (0.01 to 0.07)
Age, median (IQR)	51.6 (33.3, 67.5)	44.1 (27.2, 60.3)	-7.5 (-9.0 to -6.0)
Injury Severity Score, median (IQR)	20 (13, 26)	25 (17, 34)	5.0 (4.5 to 5.5)
Probability of Survival Score, median (IQR)	96.7 (86.3 to 99.0)	94.8 (70.0 to 98.8)	-1.9 (-3.0 to -1.0)
Severe head injury (AIS ≥4)	1237 (37.2%)	655 (38%)	0.008 (-0.02 to 0.03)
Hypotensive on arrival at the ED	1197 (36%)	741 (43%)	0.07 (0.04 to 0.09)
In Hospital Mortality, count	537 (16.1%)	277 (16.1%)	0 (-0.02, 0.02)
**Mechanism of Injury:**			
Assault, count (%)	416 (12.5%)	160 (9.2%)	-0.03 (-0.05 to -0.01)
Blunt Trauma, count (%)	3177 (94.5%)	1631 (94.6%)	0.009 (-0.004 to 0.02)
Crush, count (%)	33 (1.0%)	38 (2.2%)	0.01 (0.003 to 0.02)
Fall <2m, count (%)	929 (27.9%)	105 (6.1%)	-0.22 (-0.24 to -0.20)
Fall >2m, count (%)	665 (20.0%)	279 (16.2%)	-0.04 (-0.06 to -0.02)
Road Traffic Collision, count (%)	1079 (32.4%)	1076 (62.5%)	0.30 (0.27 to 0.33)
Other, count (%)	205 (6.1%)	66 (3.8%)	-0.02 (-0.04 to -0.01)
**Miscellaneous:**			
Total time on scene	36 minutes	40 minutes	4 minutes (2.1 to 6.7)
Time between leaving scene and arrival at ED	28 minutes	24 minutes	-4 minutes (-0.49 to 1.5) to 4.9)
Admitted to critical care	2340 (69%)	1377 (80%)	0.11 (0.081 to 0.013)
Traumatic Cardiac Arrest, count (%)	167 (4.8%)	121 (6.6%)	0.018 (0.03 to 0.004)

### Survival & mortality

There was no difference in crude mortality between the two groups (16.1%). Table 2 W and Ws statistics for each survival band in the two groups. The totals differ from those in Table 2 because some patients did not have a documented GOS and this is required to calculate the Ps.

The ECT group had 69 (4%) more survivors than predicted by the Ps17 model and the non-ECT group had 57 (-1.7%) fewer survivors than predicted. The Ws statistic aims to adjust the differences in case mix to allow comparison between groups of patients where the case mix differs. A Ws of 1 indicates one additional survivor above that predicted via Ps17 for every 100 patients. The difference between the Ws of the two groups was 5.33 (95% CI 3.63 to 7.03), equivalent to one extra survivor with ECT care for every 19 patients treated. Since it is plausible that the effects of the ECT are not consistent across all Ps bands, the ECT effect was calculated for each band.

$$\:ECT\:effect\:=\:W{s}_{ECT}\:-W{s}_{nonECT}$$

Table 2 illustrates that there was a positive ECT association in all Ps bands.

**Table 2:** Survival outcomes

	Non ECT group					ECT group					
Survival Band %	Number of patients	Expected Survivors	Actual Survivors	Difference (W)	Adj Diff (Ws)	Number of patients	Expected Survivors	Actual Survivors	Difference (W)	Adj Diff (Ws)	ECT effect
95–100	1919	1888	1869	-19	-0.512	841	827	834	7	0.430	0.942
90–95	383	355	340	-15	-0.482	181	168	170	2	0.136	0.618
80–90	289	248	240	-8	-0.2895	164	140	153	13	0.847	1.137
65–80	180	131	131	1	0.	122	89	95	6	0.417	0.417
45–65	158	87	79	-8	-0.359	146	78	90	12	0.583	0.619
25–45	195	68	7	-11	-0.311	153	54	66	12	0.432	0.743
0–25	146	17	21	4	0.113	110	16	33	17	0.639	0.526
Total	3270	2794	2737	**-57**	**-1.84**	1717	1372	1441	**69**	**3.48**	

### Morbidity

**Fig. 3:**
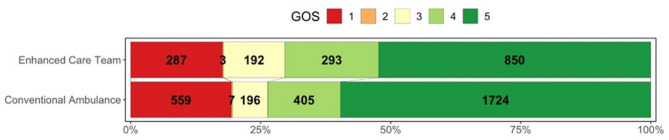
Grotta bar chart for Glasgow Outcome Scales for patients in Enhanced Care Team and Conventional Ambulance response groups (1 is the worst outcome (death) and 5 the best (good recovery))

Glasgow Outcome Scale at three months were available for 1,625 (94.2%) of the ECT and 2,891 (86.9%) of the non-ECT, the outcomes are demonstrated in (Fig. 3). In logistic regression models for dichotomised GOS (Table 3 2–3 vs. 3 and 4), univariate analysis suggested that ECT was associated with significantly reduced likelihood of good outcomes (OR 0.85; 95% CI 0.74–0.97). This analysis fails to consider the different baseline characteristics of the two groups (greater injury severity in the ECT group). After adjusting for differences in Ps17 score between the groups, multivariate logistic regression for the association between ECT and good functional outcomes demonstrated no significant difference (OR 1.18; 95% CIs 1.00-1.40).

**Table 3:** Results of univariate and multivariate logistic regression models for a poor outcome (Glasgow outcome scale 1–3), for enhanced care team presence and Ps17 survival probability score

		Odds Ratio of GOS 4–5 (95% CIs)	*p* value	Adjusted Odds Ratio of GOS 4–5 (95% CIs)	*p* value
**Service**	**Non ECT**	Reference	-	Reference	-
	**ECT**	0.85 (0.74–0.97)	0.017	1.18 (1.00-1.40)	0.058
**Ps17**	**Each 1% Increase**	1.06 (1.05–1.06)	< 0.001	1.06 (1.05–1.06)	< 0.001

**Table 4:** Critical care interventions

Interventions	Non-ECT patients *N* = 3327	ECT patients *N* = 1724	Difference in proportions (95% CI)
Total patients receiving critical care procedures (%)	1,287 (38.7)	1,381 (80.1)	-0.41 (-0.44 to -0.39)
Patients receiving critical care procedures on scene	38 (1.1)	981 (56.9)	-0.56 (-0.53 to -0.58)
Patients receiving critical care procedures in hospital	1266 (38.1)	536 (31.1)	0.07 (0.04 to 0.10)
Patients receiving advanced airway management:	997 (30.0)	1002 (58.1)	-0.28 (-0.31 to -0.25)
Intubation without drugs	38 (1.1)	43 (2.5)	-0.01 (-0.02 to -0.006)
Pre-hospital emergency anaesthetic	0	673 (39.0)	-0.39 (-0.41 to -0.37)
Rapid Sequence Intubation in hospital	959 (28.8)	286 (16.6)	0.12 (0.10 to 0.15)
Patients receiving blood products:	486 (14.6)	776 (45.0)	-0.30 (-0.33 to -0.28)
On scene only	0	266 (15.4)	-0.15 (-0.17 to -0.14)
In hospital only	486 (14.6)	304 (17.6)	-0.03 (-0.05 to -0.01)
On scene and in hospital	0	206 (11.9)	-0.12 (-0.14 to -0.11

Note, patients may have received > 1 critical care intervention, e.g. intubation and transfusion, and may have had an intervention both on scene and in hospital

### Critical care interventions

Table 4 shows the critical care interventions received by each group. Overall critical care interventions were more common in the ECT group: 80.1% of the ECT group, 38.7% of the non-ECT group (95% CI for difference 39% to 44%). Both total RSI and total blood product infusion were more common in the ECT group: 55.6% vs. 28.8% (95% CI for difference 24.0% to 29.6%) for RSI; 45.0% vs. 14.6% (95% CI for difference 27.8% to 33.0%) for blood product infusion.

**Fig. 4:**
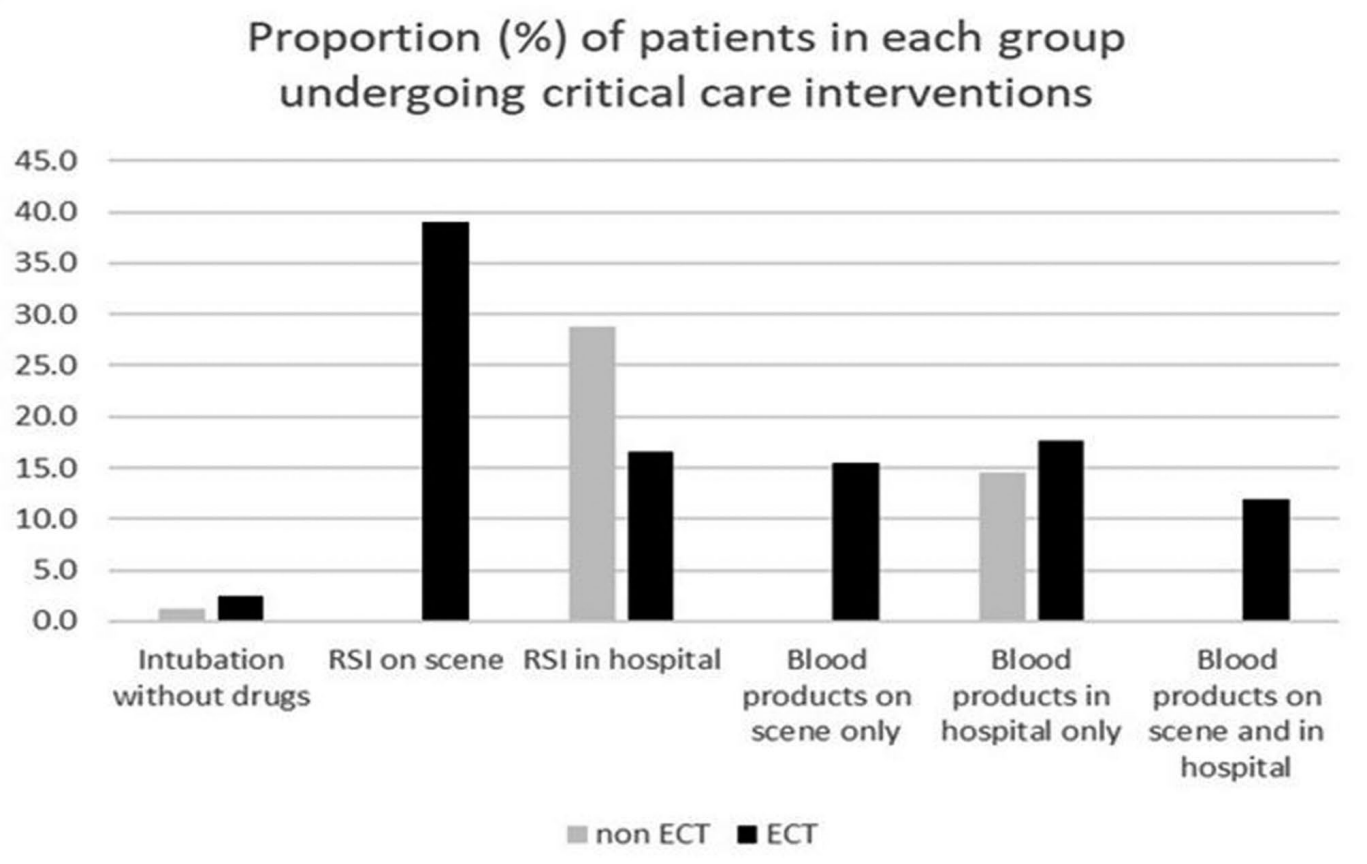
The proportion of patient in each group undergoing critical care interventions

There was a statistical difference in the mortality rate of patients who sustained a severe head injury (AIS > 4) and received a PHEA or RSI in ED for both low and high GCS groups: ECT 37% vs. non-ECT 46% for GCS 3 to 9, and 7% vs. 19% respectively, for GCS 10 to 15 (Fig. 4).

### Traumatic cardiac arrests (TCA)

**Fig. 5:**
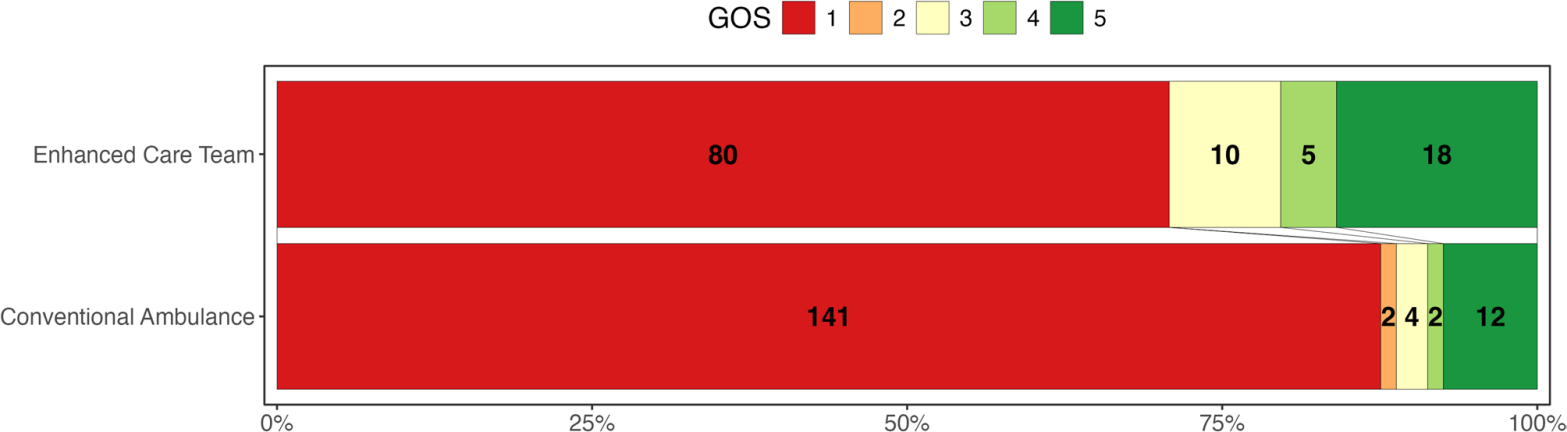
Grotta bar chart for Glasgow Outcome Scales for patients treated for Traumatic Cardiac Arrest in Enhanced Care Team and Conventional Ambulance response groups (1 is the worst outcome (death) and 5 the best (good recovery))

Of 121 cases of traumatic cardiac arrest treated by the ECT (median Ps17 45.3), 113 (93.4%) had functional outcome data at 3 months, 23 (20.4%) of these made a good recovery (GOS 4 or 5). In the conventional ambulance response group (median Ps 40.3), from 167 cases of TCA, 161 (96.4%) had follow-up functional data at 3 months. Of these, 14 (8.7%) made a good recovery (Fig. 5). The difference in survival between the two groups was statistically significant (29.2% vs. 12.4% *p* < 0.001).

## Discussion

Within its limitations as a single centre retrospective observational study, this study aimed to assess the impact of a regional ECT on mortality and morbidity of trauma patients. The key result is an association of 3.48 to 5.3 more survivors for every 100 patients receiving ECT care. There was no significant difference in the likelihood of poor outcomes by the Glasgow Outcomes Scale between the groups.

### How does this compare with previous data from the first and other studies?

This study supports previous European studies [2, 10–17]. Improvements in mortality and morbidity are likely multifactorial but may be due in part to the clinical decision making, increased resources on scene, reduced transfer time and increased number of time critical prehospital procedures delivered to address airway compromise and haemorrhage.

This study differs from previous published ECT studies by comparing matched cases using retrospective criteria and Ps banding.

### Why is there this effect?

Understanding the impact of individual critical care interventions is challenging. The effectiveness may be attributed to the comprehensive care package offered by ECTs. This includes promptly identifying injuries causing physiological abnormalities and acting with a suite of critical care bundles. The ability of ECTs as an extra resource to organise the scene and their capacity to communicate effectively with hospital trauma teams ensures timely fulfilment of the patient’s immediate requirements on arrival to the resuscitation room. Collectively, these factors may contribute to better overall outcomes.

ECTs had a measurable impact on all PS bands but the benefit on those patients with a high likelihood of survival maybe less pronounced. Patients with higher Ps bands may have sustained isolated injuries significant enough to cause physiological derangement such as hypoxia and hypotension which may affect morbidity but not Ps, hence the true ECT association may be hidden.

The results show there was a statistical difference in the mortality rate of patients who sustained a severe head injury (AIS > 4) and received a PHEA or RSI in ED. Early targeting of oxygenation and ventilation strategies by the ECT may mitigate the negative effects of secondary brain injury.

ECT case reviews of the survivors of traumatic cardiac arrest indicate that targeted interventions to treat the primary pathology causing the arrest, rather than protocolised generalised treatments, may have a positive effect. Examples include those patients with hypoxia being treated with early intubation/ ventilation and those with non-compressible torso Haemorrhage being treated with early blood products and rapid transport to hospital for damage control surgery.

### Limitations

This study is a retrospective observational study, it is subject to bias that a randomised control trial would seek to minimise. It can demonstrate only association and not causation. The prospect of conducting a randomised control trial is unlikely at this juncture as PHEM Consultant-CCP ECTs are becoming the established standard for pre-hospital critical care in the UK. Previous planned RCTs such as the HIRT trial terminated early due to slow recruitment and poor protocol compliance [17].

This work excluded patients with more minor injuries and investigated only 33% of ECT cases and 17% of ambulance trauma patients over the study period. There may have been patients missed by this study with lower ISS or single system pathology who still benefited from ECT interventions.

There may be a survival bias due to more intensive prehospital resuscitation in the ECT group meaning a higher proportion of severely injured patients arrive at hospital alive whereas equivalent patient in the non ECT group did not.

A health economics analysis is absent in this study. The authors have not measured the impact of extra survivors or the absence of detrimental morbidity using any quality adjusted life year (QALY) metric. It is therefore difficult to determine the extra costs required to produce a mortality and morbidity benefit [18].

This study analysed data from a 13- year period, changes in the relative impact of ECT and ambulance services over this time are not assessed with this methodology. Thoracotomy and pre-hospital packed red cell transfusions became an established procedure for the ECT in early 2015, 415 patients (24%) were treated by the ECT before then raising the possibility of the outcomes for those patients may have been less favourable. Further developments led to fresh plasma being available from May 2016. Patients have benefited since this time by receiving a balanced approach to transfusion.

GNAAS deliver prehospital critical care interventions, using a physician-CCP ECT. In other systems, these same procedures may be performed by paramedics independently. The observed difference may not be the staffing itself but in scope of practice. In the UK, the training, experience, and governance needed to perform these procedures safely necessitates a physician-CCP team. Further work is required to determine to what extent specific interventions delivered by ECT contribute to the improved outcomes seen in their pre-hospital presence.

### What are the implications for practice, policy, and further research?

This paper adds support to the view that ECTs such as GNAAS, may benefit selected patients with severe trauma.

#### Author recommendation one

Other prehospital services to use TARN or similar methodology to identify their impact on outcome.

#### Author recommendation two

Trauma bypass protocols should be adapted to include access to ECTs 24/7 and recommend early ECT involvement if patients have uncontrolled airways or haemorrhage.

751 patients in the non-ETC group were transported to a trauma unit rather than an MTC (22%), 229 (30%) of these patients presented to the TU’s outside of normal hours. It is plausible that this may influence outcome. At these times, trauma units are often staffed by less experienced doctors than those at Major Trauma centres and access to diagnostic imaging and damage control surgery may be limited or delayed.

#### Author recommendation three

The intelligent tasking tools used to task the ECT in daytime must be used at night such as active, passive interrogation and video streaming from scene. If the ECT were tasked to more appropriate cases this may reduce the number of secondary transfers required from trauma units.

On average the ECT is missing around 250 severe trauma cases per year (cases treated by non-ECT’s who required critical care interventions on arrival at hospital). If ECTs were tasked to all these cases one might expect one additional one survivor each month. Over 942/3327 28% of these missed cases occur during the hours of 20:00–08:00, NEAS are currently developing a critical care desk during this period. The profile of severe trauma without ECT and using advanced paramedics merits further research.

#### Author recommendation four

An adaption to the ECT tasking process must be made to identify more falls, reaching more of these patients with the aim of improving outcomes.

The mechanism of injury differed between the two groups. Twice the numbers of road traffic collisions were in the ECT group, while five times the number of falls were seen in the non-ECT group. GNAAS ECT has specific tasking criteria, significant falls are much more difficult to identify compared with road traffic collisions. Future research may consider strategies to improve ECT tasking to falls.

#### Author recommendation five

TCA should be included within future probability of survival models.

TARN excludes prehospital traumatic arrest as a factor in determining Ps. The Ps of these patients may be overestimated as two patients of a similar age, ISS and GCS may have the same Ps but different outcomes due to the physiological effects of a traumatic cardiac arrest. Many of these patients are young, have single system injuries (low ISS) a high initial GCS (producing a high Ps) but then deteriorate into traumatic cardiac arrest often dying in the first 24 hours of their hospital stay. This may explain the decreased association of the ECT in higher survival bands. It is possible that if TCA was included in the Ps calculation the outcome benefits of ECT care would be even greater.

## Conclusions

This study demonstrates improved outcomes in major trauma with pre-hospital enhanced care team attendance with an additional 3.48 to 5.3 survivors per 100 casualties without any detriment in morbidity. Enhanced care teams may benefit selected patients with severe trauma.

## Data Availability

No datasets were generated or analysed during the current study.
